# Buffalo Medical College

**Published:** 1880-11

**Authors:** 


### BUFFALO MEDICAL COLLEGE.

THE session for 1880–81 opened on the 6th of October, with a larger class of students than have attended any previous session. The didactic lectures are given daily in regular course at the college, and in consequence of the continued illness of Prof. Miner, the department of clinical surgery has been arranged so that Prof. Moore delivers lectures on Wednesdays, at the General Hospital, while Dr. D. W. Harrington has been invited to take clinical surgery, on Saturdays, at the Hospital of the Sisters of Charity. This is a very satisfactory arrangement, and will ensure excellent clinical instruction. We congratulate the college in their selection of Dr. Harrington, whose energy and zeal in his profession has met this deserved recognition. A yet more generous sprinkling of youth in other departments would not diminish either its well-earned reputation, or the literary and scientific excellence of the instruction which the profession seek in our medical college.

